# Interleukin-1β induces trained innate immunity in human hematopoietic progenitor cells *in vitro*

**DOI:** 10.1016/j.stemcr.2024.09.004

**Published:** 2024-11-07

**Authors:** Daniela Flores-Gomez, Willemijn Hobo, Diede van Ens, Elise L. Kessler, Boris Novakovic, Nicolaas P.M. Schaap, Wim H.C. Rijnen, Leo A.B. Joosten, Mihai G. Netea, Niels P. Riksen, Siroon Bekkering

**Affiliations:** 1 Department of Internal Medicine, Radboud University Medical Center, 6525 GA Nijmegen, Gelderland, the Netherlands; 2 Department of Laboratory Medicine, Laboratory of Hematology, Radboud University Medical Center, 6525 GA Nijmegen, Gelderland, the Netherlands; 3 Laboratory for Experimental Cardiology, Department of Cardiology, University Medical Center, Utrecht, 3584 CX Utrecht, the Netherlands; 4 Murdoch Children’s Research Institute and Department of Pediatrics, University of Melbourne, Royal Children’s Hospital, Parkville, VIC 3052, Australia; 5 Department of Hematology, Radboud University Medical Center, 6525 GA Nijmegen, Gelderland, the Netherlands; 6 Department of Orthopedics, Radboud University Medical Center, 6525 GA Nijmegen, Gelderland, the Netherlands; 7 Department of Medical Genetics, Iuliu Hațieganu University of Medicine and Pharmacy, Cluj-Napoca 400347, Romania; 8 Department of Immunology and Metabolism, Life and Medical Sciences Institute, University of Bonn, 53115 Bonn, Germany

**Keywords:** hematopoietic progenitor cells, bone marrow, IL-1β, monocytes, macrophages, trained immunity

## Abstract

Innate immune cells can develop a long-lasting hyperresponsive phenotype, termed trained immunity, mediated by epigenetic and metabolic reprogramming. In mice, exposure to Bacille Calmette-Guérin (BCG), β-glucan, or Western diet induces trained immunity by reprogramming hematopoietic progenitor cells (HPCs), through interleukin-1β (IL-1β) signaling in the bone marrow (BM). We investigated whether IL-1β induces trained immunity in primary human BM-derived HPCs *in vitro*. We exposed human BM-derived HPCs to IL-1β for 4 h. HPCs were expanded and differentiated into monocytes followed by functional and transcriptomic characterization. IL-1β-exposed HPCs showed higher granulocyte-macrophage colony-forming units. The monocyte offspring produced more tumor necrosis factor (TNF) and IL-1β after restimulation with lipopolysaccharide (LPS) and Pam3Cys and is metabolically more active. Transcriptomic analysis showed upregulation of key atherogenic and inflammatory pathways. In conclusion, brief exposure of human BM-derived HPCs to IL-1β *in vitro* induces a trained immunity phenotype.

## Highlights

• Exposure of HPCs to IL-1β leads to production of inflammatory monocytes
• HPC-derived trained monocytes have increased cytokine production
• This is associated with increased glycolysis and oxidative phosphorylation
• IL-1β induces innate immune memory in human bone marrow HPCs *in vitro*

## Graphical abstract

**Figure:**
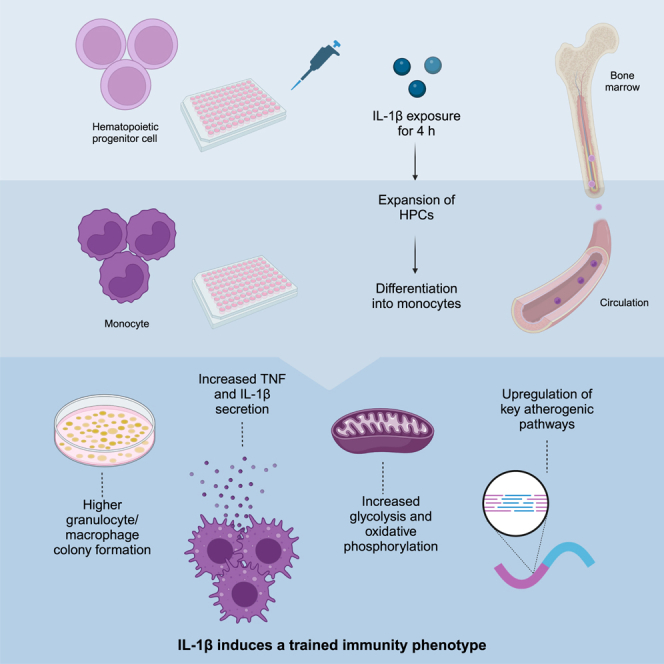

## Teaser

Flores-Gomez et al. demonstrate that brief exposure of human BM-derived HPCs to IL-1β *in vitro* induces long-term pro-inflammatory effects, characterized by increased CFU-GM colony formation and increased pro-inflammatory cytokine production capacity of IL-1β-exposed HPC-derived monocytes. These memory effects are indicative of trained innate immunity.

## Introduction

The innate immune system can develop a long-lasting pro-inflammatory phenotype after brief exposure to microorganisms or endogenous substances, such as modified lipoproteins, glucose, urate, or danger-associated molecular patterns (Bekkering et al., 2021). This phenomenon is called trained immunity, is mediated by epigenetic and metabolic reprogramming, and is characterized by an increased cytokine production capacity (Domínguez-Andrés et al., 2023).

In addition to mature immune cells, such as monocytes, trained immunity can also occur in the bone marrow progenitor cells, which is called “central trained immunity” (Riksen et al., 2023). This explains the observation that, after subcutaneous administration of Bacille Calmette-Guérin (BCG), a potent inducer of trained immunity, trained monocytes are present in the human circulation up to one year, despite the short half-life of circulating monocytes (Kleinnijenhuis et al., 2014). In mice, short-term exposure to BCG, β-glucan, or Western-type diet induces trained immunity by epigenetic reprogramming of hematopoietic progenitor cells (HPCs). Murine studies demonstrated that BCG promotes proliferation of HPCs and confers protection against other infections (Kaufmann et al., 2018). This has also been shown in studies in humans, in whom bone marrow HPCs showed functional and transcriptional reprogramming 3 months after BCG vaccination (Cirovic et al., 2020). Mitroulis et al. (2018) demonstrated that β-glucan induced HPC reprogramming via interleukin-1β (IL-1β) signaling in the bone marrow. The trained HPCs were characterized by increased proliferation, and myeloid skewing. In addition to infectious stimuli, 4 weeks of a Western-type diet in *Ldlr*−/− mice induced a similar effect resulting in enhanced stem cell proliferation and immune response, which was dependent on NLRP3 inflammasome activation and IL-1β signaling (Christ et al., 2018). Bone marrow myeloid reprogramming has also been studied in humans in the context of atherosclerotic cardiovascular diseases. Patients with established coronary artery disease have transcriptionally reprogrammed HPCs and increased cytokine production capacity (Noz et al., 2020). Similar findings were reported for patients with familial hypercholesterolemia (Stiekema et al., 2021). A key role for IL-1β in cardiovascular diseases has been shown by the observation that the anti-IL-1β antibody canakinumab lowers future cardiovascular disease risk (Ridker et al., 2017).

Based on these published data, we hypothesize that also in humans, IL-1β induces HPC-trained immunity. To test this, we designed an *in vitro* model to study trained immunity in human bone-marrow-derived HPCs and studied the effects of brief exposure to IL-1β. We assessed the functional and transcriptional parameters of trained HPC-derived monocytes and macrophages. Additionally, we performed a colony formation unit to investigate the proliferation and differentiation capacity of trained cells. Our results will help to understand how IL-1β signaling can have prolonged pro-inflammatory effects on the innate immune system.

## Results

### IL-1β induces a shift toward myeloid cell proliferation

**Figure 1:**
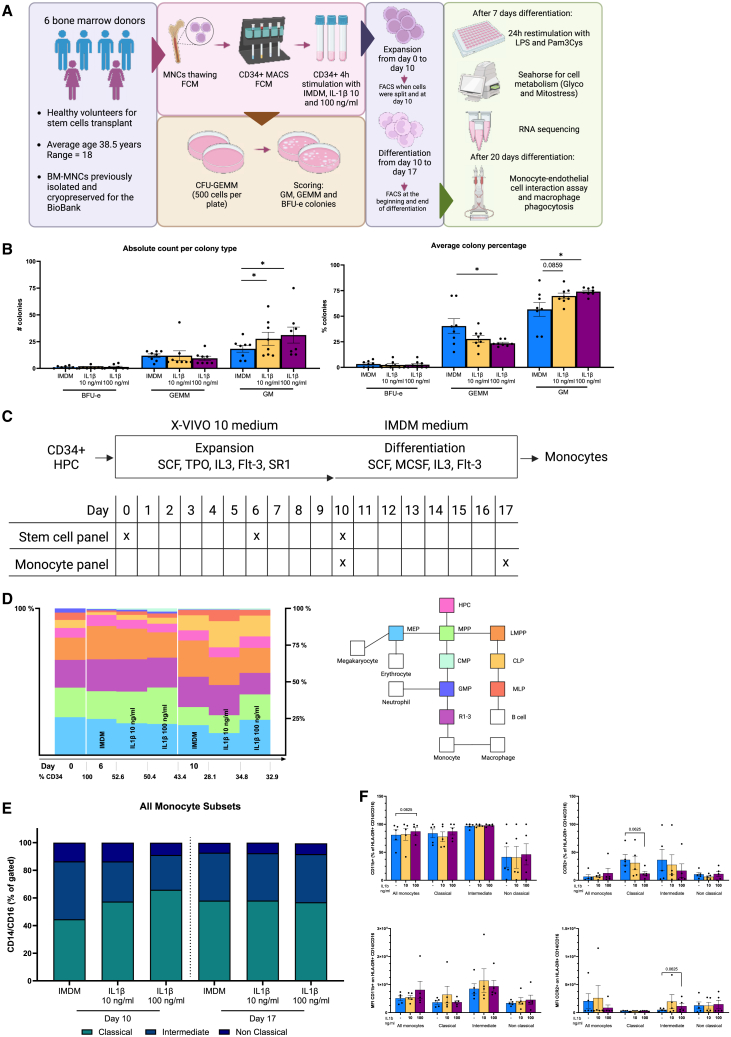
Schematic overview, proliferation and differentiation during expansion and differentiation of HPCs (A) Schematic overview of the experimental design. (B) Proliferation and differentiation capacity of human bone marrow hematopoietic progenitor cells (HPCs). Number and percentages of colonies counted after 14 days of incubation of control (IMDM) and IL-1β -exposed HPCs (IL-1β 10 and 100 ng/mL). 500 HPCs were initially seeded per condition in duplicate (*n* = 4 independent HPCs donors, Wilcoxon matched-pairs signed-rank test, ^∗^*p* < 0,05 compared to IMDM control). (C) Schematic overview of the flow cytometry panels to identify stem cell progenitor populations during expansion and mature cells during differentiation. (D) Progenitor populations in controls and IL-1β-exposed cells (10 and 100 ng/mL) at day 0, day 6, and day 10 of expansion (*n* = 4 independent HPC donors, Wilcoxon matched-pairs signed-rank test, differences were not significant). (E) Monocyte subsets identified at the beginning and end of differentiation of HPCs with M-CSF in control and cells exposed to IL-1β (10 and 100 ng/mL). IL-1β induces an increase of classical monocytes at the beginning of the differentiation (*n* = 5 independent HPC donors, Wilcoxon matched-pairs signed-rank test, differences were not significant). (F) CD11b and CCR2 activation markers expression and median fluorescence intensity (MFI) in the different bone-marrow-derived monocyte subsets after 7 days of differentiation. (*n* = 5 independent HPC donors, Wilcoxon matched-pairs signed-rank test). See also Figures S1–S3.

To study the effect of IL-1β on HPC proliferation, we exposed isolated CD34^+^ cells to IL-1β for 4 h and subsequently performed a colony-forming unit (CFU) assay (Figure 1A).

Exposure of HPCs to IL-1β increased myeloid cell production in CFU assay in a dose-dependent manner. Both the absolute colony count and the colony percentage of the granulocyte-macrophage (GM) population (Figure 1B) were significantly higher in the IL-1β-exposed conditions compared to the Iscove’s modified Dulbecco’s medium (IMDM) control cells.

### No effect of IL-1β on monocyte differentiation

After 4-h stimulation of HPCs with IL-1β, cells were expanded for 10 days and differentiated into monocytes for another 7 days. During the culture time, the cells had similar morphology and expansion and differentiation rates (Figure S1). During the expansion and differentiation time, flow cytometry was performed to understand the effect of IL-1β on the lineage of differentiation of HPC (Figure 1C). As shown in Figure 1D, progenitor populations changed over the course of expansion, but did not significantly differ between trained and untrained conditions. We subsequently defined the three monocyte subsets with flow cytometry during the 7 days of differentiation. A sequential ontogeny scenario describes that classical monocytes can differentiate into intermediate monocytes and, finally, non-classical monocytes (Ruder et al., 2023). Although there was a trend toward more classical monocytes after IL-1β (100 ng/mL) exposure at day 10, there were no differences on day 17 (Figure 1E). Neither were there any significant differences in the surface expression of CD11b and CCR2 (Figure 1F).

### IL-1β increases HPC-derived monocyte cytokine production and cellular metabolism

**Figure 2:**
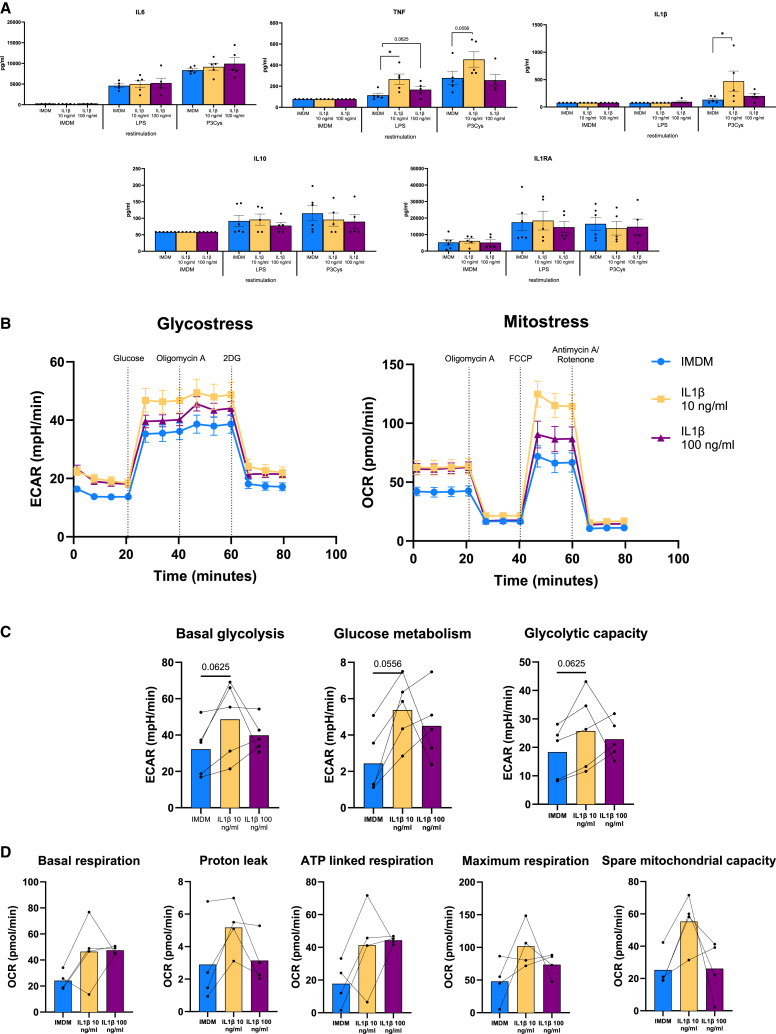
Short exposure of HPCs to IL-1β augments cytokine production capacity and affects cellular metabolism of bone-marrow-derived monocytes HPCs were exposed to IL-1β (10 and 100 ng/mL) for 4 h. After 10 days of expansion and 7 days of differentiation. (A) Cells were restimulated with LPS and Pam3Cys for 24 h and IL-6, TNF, IL-1β, IL-10, and IL-1RA production were measured (*n* = 5 independent HPC donors, ^∗^*p* < 0.05, Wilcoxon matched-pairs signed-rank test). (B) Extracellular acidification rate (ECAR) over time during subsequent injection of glucose, oligomycin A, and 2DG. Oxygen consumption rate over time during subsequent injection of oligomycin A, FCCP, and antimycin A/rotenone (*n* = 5 independent HPC donors for glycostress test and *n* = 4 independent HPC donors for mito stress test, Wilcoxon matched-pairs signed-rank test). (C) Bar graphs with individual points of basal glycolysis, glucose metabolism, and glycolytic capacity of IL-1β-trained cells compared to control (*n* = 5 independent HPC donors, Wilcoxon matched-pairs signed-rank test). (D) Bar graphs with individual points of basal respiration, proton leak, ATP-linked respiration, maximum respiration, and spare mitochondrial capacity of IL-1β-trained cells compared to control (*n* = 4 independent HPC donors, Wilcoxon matched-pairs signed-rank test).

The classical functional hallmark of trained immunity is an increased cytokine production capacity upon restimulation (Bekkering et al., 2021; Tercan et al., 2021). After short exposure of CD34^+^ cells to IL-1β, cells were expanded for 10 days and differentiated into monocytes for 7 days. Then, HPC-derived monocytes were restimulated with TLR2 and 4 agonists to assess cytokine response. Tumor necrosis factor (TNF) production after lipopolysaccharide (LPS) stimulation and IL-1β production after Pam3Cys stimulation were significantly increased in the monocytes derived from HPCs after IL-1β exposure (10 ng/mL). The anti-inflammatory cytokines IL-10 and IL-1Ra did not show any increase in the IL-1β situation (Figure 2A).

The functional hyperresponsiveness of trained cells is accompanied by an increase in glycolysis and oxidative phosphorylation (Domínguez-Andrés et al., 2023; Riksen et al., 2023). To explore the effects of IL-1β on these metabolic processes, we performed Seahorse analysis of HPCsderived monocytes. This revealed that glycolysis (extracellular acidification rate), as well as mitochondrial respiration (oxygen consumption rate [OCR]), displays a trend to be higher in IL-1β-trained HPC-derived monocytes compared to untrained controls (Figure 2B). This upregulation was also observed in various individual parameters of glycolysis and mitochondrial respiration such as basal glycolysis, maximum glycolytic capacity, basal respiration, and maximum mitochondrial respiration (Figures 2C and 2D). It is remarkable that the changes appeared more pronounced after exposure to 10 ng/mL IL-1β than after exposure to 100 ng/mL, which aligns well with the cytokine production capacity in both conditions (Figures 2A and 2B).

### Effects of IL-1β on HPC-derived monocyte RNA transcription

**Figure 3:**
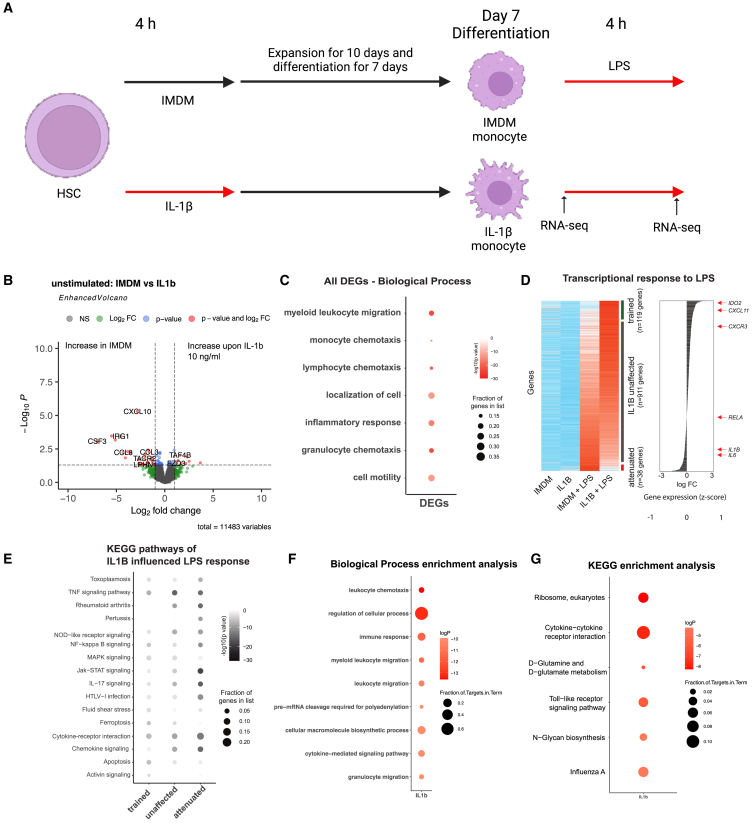
Short exposure to IL-1β 10 ng/mL and restimulation with LPS induce transcriptional changes in HPC-derived monocytes (A) Schematic overview of the protocol used to collect RNA samples of IL-1β-trained cells. Magnetically sorted monocytes were stimulated with 10 ng/mL LPS for 4 h. Samples were collected before and after LPS restimulation for RNA-seq. (B) Volcano plot showing up and down-regulated genes between IMDM and IL-1β-exposed monocytes before LPS exposure. *p* values were adjusted for multiple comparisons. (C) Top biological pathways enriched in the differentially expressed gene (DEG) list, according to *p* value and fraction of DEG present. (D) Transcriptional response to LPS restimulation. Genes were clustered in 3 groups: IL-1β trained (119 genes), IL-1β attenuated (911 genes), and unaffected (38 genes). (E) KEGG pathway analysis showing the influence of IL-1β upon LPS restimulation in the trained, IL-1β-attenuated, and unaffected clusters according to *p* value and fraction of DEG present. For all the analysis, *n* = 3 independent HPC donors was used. (F) Biological Process (BP) enrichment and (G) KEGG pathway enrichment analysis on the 371 dynamic genes (*p* < 0.05, FC > 1.2), in response to at rest or after LPS exposure, relative to IMDM macrophages.

To investigate the effects of IL-1β exposure on transcriptional changes in HPC-derived monocytes, we performed RNA sequencing on isolated HPC-derived monocytes exposed to IL-1β 10 ng/mL (4 h), followed by expansion (10 days), differentiation (7 days), and stimulation with LPS for 4 h (Figure 3A). Following expansion and differentiation, IMDM and IL-1β monocytes display limited differences in gene expression profile before restimulation (Figure 3B). Some of these genes are involved in relevant biological processes as seen in Figure 3C, such as myeloid leukocyte migration, inflammatory response, and granulocyte chemotaxis.

Next, we analyzed gene expression following LPS exposure, to determine how IL-1β exposure influenced the monocyte response to secondary stimulation (Figure 3D). The heatmap was ranked by the effect of IL-1β on LPS response, with “trained” (119 genes), “unaffected” (911 genes) and “attenuated” (38 genes) responses by IL-1β exposure of HPCs, based on both conditions before and after LPS restimulation. Interestingly, *IL1B* and *IL6* genes were clustered as IL-1β-unaffected genes, contrary to what we observed in the cytokine production capacity of these trained HPC-derived monocytes. Similarly, pathways like TNF signaling, Jak-STAT, and chemokine signaling were more represented in the attenuated or unaffected genes (higher fractions of differentially expressed genes present), while the “trained” gene group was enriched for apoptosis and ferroptosis (Figure 3E).

We further looked at the effect of IL-1β exposure on the expression of epigenetic regulators, by downloading the list of 773 epigenetic modifiers from the FANTOM Consortium EpiFactors database (Medvedeva et al., 2015). We found that, of these 773 epigenetic factors, 17 were in the list of 371 dynamic genes (*p* < 0.05, FC > 1.2) in response to IL-1β at rest or after LPS exposure, relative to IMDM macrophages (see Table 1). In general, epigenetic factors were slightly more likely to be dynamic (2.2% vs. 1.8%) compared to all genes. This includes *TET2*, which is less expressed in IL-1β-exposed cells, both at rest and after LPS exposure.

**Table 1:** Dynamic epigenetic modifier genes

HGNC_symbol	UniProt_AC	Function	Row.names	log2FoldChange	*p* value	uns_IMDM_CPM_mean	uns_IL1_CPM_mean	LPS_IMDM_CPM_mean	LPS_IL1_CPM_mean
*RB1*	P06400	Chromatin_remodeling_Histone_modification_write	ENSG00000139687	0.4255035	0.02512911	30.10721	35.07385	17.32411	23.93343
*EPC1*	Q9H2F5	Polycomb_group_(PcG)_protein	ENSG00000120616	0.5183384	0.03820138	17.91373	19.09535	16.88228	24.48446
*PADI2*	Q9Y2J8	Histone_modification	ENSG00000117115	−0.5777374	0.01846565	27.86381	28.84332	22.57345	14.95674
*ATXN7*	O15265	Histone_modification_write_cofactor	ENSG00000163635	0.4969222	0.02535242	21.01068	21.27799	16.05089	22.54999
*CBLL1*	Q75N03	RNA_modification	ENSG00000105879	−0.3146693	0.03907087	63.81039	63.00588	61.67063	49.84006
*LEO1*	Q8WVC0	Histone_modification_write_cofactor	ENSG00000166477	−0.6266516	0.03412533	59.58235	54.63181	45.91457	30.6547
*SUV39H1*	O43463	Histone_modification_write_Histone_modification_write	ENSG00000101945	−0.389872459	0.048444966	22.41103926	20.22470767	19.85635302	15.28346169
*ZNF687*	Q8N1G0	Histone_modification_erase_cofactor	ENSG00000143373	−0.7940353	0.03142792	10.3149	9.052185	11.01229	6.06947
*PELP1*	Q8IZL8	Histone_modification_read_Histone_modification_write_cofactor	ENSG00000141456	0.9922168	0.03417424	1.905422	1.664677	1.383398	2.641318
*TLK1*	Q9UKI8	Histone_modification_write	ENSG00000198586	−0.7335763	0.0185721	169.5784	144.4192	118.6951	68.78121
*TET2*	Q6N021	DNA_modification	ENSG00000168769	−0.4899147	0.003111455	182.861	151.5804	137.961	98.30065
*GSG2*	Q8TF76	Histone_modification_write	ENSG00000177602	−1.098119	0.005134638	5.759229	4.306792	4.360039	2.105806
*DZIP3*	Q86Y13	Histone_modification_write	ENSG00000198919	0.8927027	0.01575299	6.091953	4.536273	3.760852	6.784402
*SMARCAL1*	Q9NZC9	Chromatin_remodeling	ENSG00000138375	−0.657767344	0.042628458	9.609686796	6.122826505	5.696649864	5.011683018
*USP49*	Q70CQ1	Histone_modification_erase	ENSG00000164663	−0.79180809	0.01000105	8.15194296	4.521924333	6.678193826	7.186339536
*TAF6*	P49848	Histone_chaperone	ENSG00000106290	−0.869155484	0.042491593	6.090293369	3.246512457	3.758781191	2.431951576
*APOBEC3A*	P31941	DNA_modification_RNA_modification	ENSG00000128383	−2.046492087	0.008185171	4.947446991	1.683743614	3.208114535	2.842598104

Seventeen genes from the EpiFactors database of 773 epigenetic modifiers were in the list of 371 dynamic genes (*p* < 0.05, FC > 1.2) in response to IL-1β at rest or after LPS exposure, relative to IMDM macrophages.

Finally, we also performed pathway analysis on the 371 dynamic genes in response to at rest or after LPS exposure, relative to IMDM macrophages (see Figures 3F and 3G). Gene Ontology Biological Process analysis revealed an enrichment in “regulation of cellular processes” and “cellular macromolecule biosynthetic process,” suggesting involvement in metabolic processes. Moreover, enrichment in “leukocyte chemotaxis,” “immune response,” and “leukocyte migration” fits with our functional results suggestive of increased adherence to endothelial cells (ECs). Kyoto Encyclopedia of Genes and Genomes (KEGG) pathway analysis showed enrichment in “cytokine-cytokine receptor interaction” and “Toll-like receptor signaling pathway,” which again align with the functional hyperresponsive cytokine production after Toll-like receptor stimulation.

### Exploratory studies of IL-1β on macrophage phagocytosis and EC interactions

**Figure 4:**
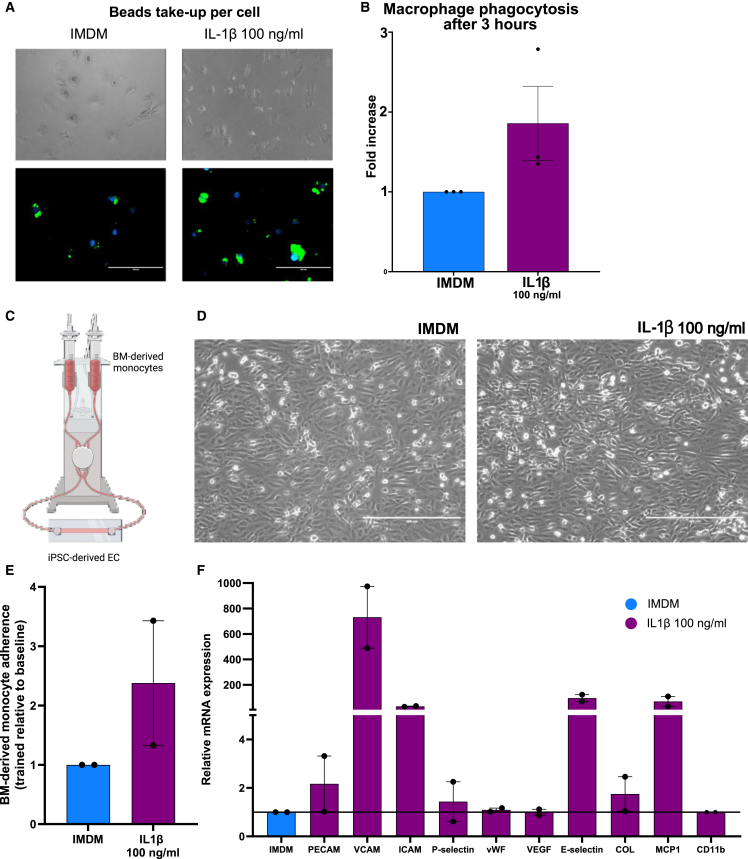
IL-1β induces increased phagocytosis and a suggestively higher endothelial cell interaction with HPC-derived monocytes (A) After 4 h of HPC exposure to IL-1β 100 ng/mL or untrained control, HPC-derived monocytes were differentiated into macrophages. After that, we added fluorescent Latex beads for 3 h and after washing made images with the EVOS microscope to measure phagocytosis capacity. Macrophages are visualized with 10× magnification, scale bar, 200 μm. (B) Phagocytosis rate quantification of IL-1β-trained macrophages compared to untrained control (*n* = 3 independent HPC donors, Wilcoxon matched-pairs signed-rank test). (C) General overview of the IBIDI system to measure endothelial cell (EC)-monocyte interaction. (D) Visualization of HPC-derived monocytes attached to EC of IL-1β-trained cells compared to the control (10× magnification, scale bar, 400 μm). (E) Relative adherence of IL-1β 100 ng/mL trained monocytes to EC relative to baseline (*n* = 2 independent HPC donors). (F) Relative mRNA expression of cell adhesion molecules and endothelial activation markers indicating various stages of leukocyte extravasation (*n* = 2 independent HPC donors).

To further characterize and determine the change in functionality of IL-1β-trained cells, we differentiated HPC-derived monocytes into macrophages and measured their phagocytic capacity after 3 h. As seen in Figure 4A, IL-1β (100 ng/mL)-trained HPC-derived monocyte-derived macrophages have higher phagocytic capacity compared to untrained control. Phagocytosis was quantified by measuring the beads intake (green) overlayed with the presence of an existing cell (blue) (Figure 4B).

We subsequently assessed the interaction and adherence of HPC-derived-monocytes to induced pluripotent stem cell (iPSC)-derived ECs in a continuous flow system (Figure 4C). After 3 h of flow, IL-1β-trained HPC-derived monocytes showed a higher adherence to ECs compared to untrained controls (Figures 4D and 4E). Subsequently, we measured the gene expression of various markers of endothelial and vascular dysfunction in the cells (iPSC-EC and attached HPC-derived monocytes) used in the system, and we observed that key genes such as vascular cell adhesion molecule, intercellular cell adhesion molecule, and E-selectin are upregulated after interaction with IL-1β-trained HPC-derived monocytes (Figure 4F).

As these experiments were only performed in two donors, we did not perform any statistical testing.

## Discussion

In this study, we aimed to test our hypothesis that brief exposure of human HPCs to IL-1β would lead to long-term pro-inflammatory effects by trained immunity, characterized by myeloid skewing and production of monocytes and macrophages with persistent pro-inflammatory characteristics. Indeed, after brief exposure of HPCs to IL-1β for only 4 h, there was an increased colony formation of GM colonies after 14 days. On a functional level, exposure to IL-1β augmented TNF and IL-1β secretion by HPC-derived monocytes upon restimulation with LPS and Pam3Cys 17 days later, respectively, whereas the production of the anti-inflammatory cytokines IL-10 and IL-1Ra remains unaffected. This hyperresponsiveness was accompanied by a trend of increased glycolysis and oxidative phosphorylation.

Accumulating experimental evidence points to a central role of trained immunity in the pathophysiology of several diseases, including atherosclerosis (Riksen et al., 2023). Mechanistically, IL-1β signaling has been identified in experimental murine models as a key mechanism in the bone marrow niche, responsible for trained immunity triggered by β-glucan (Mitroulis et al., 2018) as well as by Western diet (Christ et al., 2018). In human cells *in vitro*, Arts et al. previously described that 24 h exposure of human monocytes to 1 and 10 ng/mL of IL-1β induces trained immunity (Arts et al., 2018).

In patients with an acute myocardial infarction, plasma and bone marrow IL-1β concentrations rapidly increase (Guillén et al., 1995; Sreejit et al., 2022), which could be a trigger for the trained immunity that develops after myocardial infarction (Dong et al., 2024).

Based on these earlier studies, we hypothesized that also in human HPCs, brief exposure to IL-1β triggers trained immunity. Indeed, we observed that pro-inflammatory cytokine production, which is the major functional hallmark of trained immunity, is increased in HPC-derived monocytes after brief IL-1β exposure. RNA sequencing analysis of LPS-restimulated monocytes showed an enrichment in ferroptosis and apoptosis pathways, indicating processes which are critical in the development and progression of atherosclerotic plaques (Ouyang et al., 2021). For some parameters, including cytokine production, but not proliferation, the effect of IL-1β was stronger in 10 ng/mL than in 100 ng/mL. Interestingly, this dose dependency with lower concentration, giving rise to a stronger trained-immunity response, is not new. We have previously reported for both LPS and Pam3Cys (TLR2 agonist) that the trained immunity effect was stronger with lowering of the concentrations from 100 ng/mL to 0.1 pg/mL for LPS and from 100 μg/mL to 100 pg/mL for Pam3Cys (Ifrim et al., 2014).

We did not fully elucidate the mechanisms through which IL-1β induced memory effects. In general, trained immunity is dependent on profound metabolic and epigenetic reprogramming (Fanucchi et al., 2021). We previously showed that oxLDL- and β-glucan-trained cells are characterized by increased glycolysis and oxidative phosphorylation (Domínguez-Andrés et al., 2023; Tercan et al., 2021). This fits with our current finding that monocytes derived from IL-1β-exposed HPCs present increased cell metabolism (glycolysis and oxidative phosphorylation), although this did not reach statistical significance due to limited sample size. Pathway analyses of differentially expressed genes in the IL-1β-trained cells also showed enrichment in metabolism-related pathways, but these need further evaluation in future studies. Although we did not perform any epigenomic assays, we explored epigenetic genes among the list of IL-1β-induced dynamic genes and observed a slightly higher percentage of dynamic genes compared to all genes. Among these dynamic genes was *TET2*, which is involved in DNA demethylation. It is known that BCG-induced trained immunity is associated with changes in DNA methylation (Bannister et al., 2022). In addition, it is known in experimental models that myeloid *TET2* deficiency is associated with increased IL-1β production, and with enhanced atherosclerosis development (Fuster et al., 2017). How exactly lower *TET2* expression leads to the hyperresponsive trained phenotype needs further investigation. Lavillegrand et al. recently showed that high-fat diet-induced trained immunity developed in the bone marrow niche due to increased IL-1β production by bone marrow granulocytes, which further stimulated myeloid skewing (Lavillegrand et al., 2024). It would be interesting to further investigate IL-1β production by progenitor cells and their offspring cells in the bone marrow.

An unexpected finding was that in the trained cells, the protein concentrations on TNF and IL-1β in the supernatants after LPS stimulation were higher, in contrast to the mRNA of these proteins in the sequencing analysis. Interestingly, we had the same observation for training with β-glucan (Jentho et al., 2021; Novakovic et al., 2016). Specifically, despite observing increased TNF secretion in β-glucan and heme-trained macrophages, we did not find a difference in H3K27ac levels at the TNF promoter or increased TNF expression following restimulation in beta-glucan and heme-trained macrophages. One explanation is that the innate immune training signature in the trained macrophages involves the up-regulation of mechanisms resulting in cytokine release (e.g., lysosome maturation, Warburg effect) but not cytokine gene priming. This is in contrast to LPS-induced tolerance, which indeed attenuates both TNF secretion and RNA expression.

The main parameters in our study are proliferation and cytokine production capacity. In addition to that, we performed some exploratory studies on phagocytosis and on adhesion to iPSC-derived ECs, which are both key aspects of atherosclerosis pathophysiology We observed that training with IL-1β tends to increase monocyte attachment to iPSC-derived ECs. In addition, relative mRNA expression of key cell adhesion molecules was suggestively higher in the iPSC-derived ECs that encountered IL-1β-trained HPC-derived monocytes. These findings suggest that the monocytes that derive from trained HPCs can accelerate atherosclerosis formation by augmented attachment to ECs. These findings, however, are preliminary and need future validation, since we only performed these complex experiments for two donors, and therefore could not perform statistical testing.

After differentiation into macrophages within the arterial wall, phagocytosis is an important mechanism modulating further plaque growth, by foam cell formation and by engulfing necrotic neighboring cells in a process called efferocytosis (Kojima et al., 2017). It appeared that macrophages derived from IL-1β-trained HPCs have more phagocytic capacity compared to untrained controls.

The strength of our study is that we performed a very extensive panel of functional, flow cytometric, and transcriptional parameters to characterize the monocytes and macrophages that derive from the HPCs. We show long-lasting and biologically relevant changes even after only 4 h of HPC exposure to IL-1β. This strongly underscores the relevance of previous preclinical studies on IL-1β-induced trained immunity for the human situation. There are also some limitations to our study. First, all the experiments were performed *in vitro* in isolated HPCs. Even though we made use of cytokines and growth factor mimicking the bone marrow niche, there are some local niche factors that could alter cell function *in vivo*. Second, for some of the atherosclerosis-related functional characterization, we only performed experiments in two donors and only for one IL-1β concentration, which precluded us from formal statistical testing. This makes the results obtained exploratory and would need additional experiments and higher donor number in future studies.

In conclusion, our results convincingly demonstrate that a brief exposure of human HPCs to IL-1β induces trained immunity, which results in the formation of monocytes and macrophages with pro-inflammatory functions. These results strongly underscore the relevance of previous preclinical studies on IL-1β-induced trained immunity for the human situation and allow for the further use of this model to study trained immunity at the level of the bone marrow and to unravel mechanisms and potential therapeutics.

## Experimental procedures

### Human subjects

Bone marrow was aspirated from otherwise healthy patients undergoing orthopedic surgery and from healthy stem cell donors, which were between 30 and 48 years old. Exclusion criteria were use of immunosuppressants, recurrent infections, bone marrow malignancies/diseases, mental incapacitation, or previous radiation treatment. All donors provided written informed consent. The use of this material was approved by the Ethics Committee (Ethical Approval CMO Arnhem-Nijmegen, 2013/064) and all the experiments were performed according to the principles in the declaration of Helsinki.

### Human bone-marrow-derived mononuclear cell isolation and cryopreservation

Bone marrow mononuclear cells (BM-MNCs) were isolated by density centrifugation using Ficoll-Plaque PLUS (GE Healthcare Biosciences), followed by 3 washes with cold phosphate-buffered saline (PBS, Gibco). Mononuclear cells (MNCs) were cryopreserved in a freezing solution containing IMDM (Gibco), 1% penicillin/streptomycin (p/s, Gibco), 250 IU/mL of sodium heparin (6006501), and 7% dimethyl sulfoxide for cell culture (DMSO, AppliChem, A3672-0250) and were stored in liquid nitrogen until further use.

### Thawing of MNCs

Thawing of bone-marrow-derived MNCs was performed using DNase from bovine pancreas (Sigma) and magnesium chloride (MgCl_2_, Sigma-Aldrich, M2393-100g). See supplementary methods for details.

### CD34^+^ magnetic-activated cell sorting

BM-MNCs were centrifuged at 300G, 4°C for 10 min and the pellet was resuspended in 300 μL of magnetic-activated cell sorting (MACS) buffer containing PBS, pH 7.2, 0.5% sterile bovine serum albumin solution (BSA 30%, Merck, A9576-50ML), and 2 mM UltraPure EDTA (0.5 M, pH 8, Life Technologies). CD34^+^ HPCs were isolated from BM-MNC with MACS using a CD34 Microbead Kit according to the manufacturer’s instructions (purity >95%, data not shown) (MACS, 130-046-702, Miltenyi Biotec). After separation, cells were resuspended in supplemented IMDM and viable cells were manually counted using the trypan blue exclusion method.

### CD34^+^ cell stimulation with IL-1β

As shown in Figure 1A, HPCs were stimulated in a round-bottom 5 mL polystyrene Falcon tube (Corning, 352058) for 4 h with IMDM only as negative control and 10 and 100 ng/mL of IL-1β (R&D). After 4 h, the cells were spun down at 500 g for 10 min at room temperature, the supernatant containing the stimulus was removed, and the cells were resuspended in X-VIVO 10 serum-free hematopoietic cell medium (Lonza, BE04-380Q). Then, viable cells were manually counted using the trypan blue method.

### Proliferation and differentiation assay CFU-GEMM

CFU assay for granulocyte, erythrocyte, monocyte, and megakaryocyte (CFU-GEMM) was performed by culturing 500 cells of previously stimulated HPCs or controls in methylcellulose medium (STEMCELL Technologies, GF H84435) in 35 mm polystyrene Petri dishes (Corning, 430165). The plates were seeded in duplicate and incubated for 14 days at 37°C and 5% CO_2_. A CFU assay can derive colonies including blast forming unit erythrocyte (BFU-e), GM, and granulocyte/erythrocyte/monocyte/megakaryocyte (GEMM). After 14 days, BFU-e, GM, and GEMM colonies were scored and counted using a gridded scoring plate in an inverted microscope using high-power focus (Leica DMi1) according to the manufacturer.

### CD34^+^ cell expansion

A total of 5 × 10^4^ previously stimulated or unstimulated HPCs were seeded per well in a flat-bottom 24-well plate (Sarstedt) and were expanded in X-VIVO 10 medium supplemented with 4% FCS, 1% p/s, human stem cell factor 50 ng/mL (SCF, 130-093-991, Miltenyi Biotec), human thrombopoietin 15 ng/mL (130-094-745, Miltenyi Biotec), human IL-3, 30 ng/mL (130-093-909, Miltenyi Biotec), human FMS-like tyrosine kinase 3 ligand 30 ng/mL (Flt-3L, 130-096-474, Miltenyi Biotec), and StemRegenin-1 aryl hydrocarbon receptor agonist 2 μM (#72342, Stem Cell Technologies). Medium was changed on day 3 and day 6 of proliferation. Cells were split at ∼75%–80% confluence and re-seeded in a concentration of 5 × 10^4^ cells per well, allowing them to be in culture for at least 2 days before ending the proliferation phase.

Cells were detached using warm Versene (Life Technologies) for 5 min and cold PBS + 2 mM EDTA washes. Cells were then centrifuged at 300G, 10 min at 4°C, and resuspended in IMDM + 1% p/s. Viable cells were counted using the trypan blue exclusion method.

### CD34^+^ cell differentiation into monocytes

After 10 days of expansion, cells were differentiated into monocytes for 7 days using IMDM medium supplemented with 10% FCS, 1% p/s, SCF 25 ng/mL, human macrophage colony stimulating factor 30 ng/mL (M-CSF, 130-096-491, Miltenyi Biotec), IL-3 30 ng/mL, and Flt-3L 30 ng/mL. Medium was changed on day 3 of differentiation. Cells were detached using warm Versene for 5 min and cold PBS + 2 mM EDTA washes. Cells were then centrifuged at 300G, 10 min at 4°C, and resuspended in IMDM + 1% p/s. Viable cells were counted using the trypan blue exclusion method.

Differentiation was continued for part of the cells for 13 more days to increase the number of cells, using supplemented IMDM as mentioned before. After 20 days of differentiation in total, cells were detached and used for IBIDI flow experiments, macrophage differentiation, macrophage polarization, and phagocytosis.

### Flow cytometry

Flow cytometry was performed at several time points (Figure 1C): during expansion of HPCs and their differentiation into monocytes. During expansion, stem cell markers relevant to progenitor populations were measured at day 0, 6, and 10. Cells were stained with monoclonal antibodies cluster of differentiation (CD)117, CD19, CD38, CD10, CD45RA, CD34, CD123, CD45, CD90, and live/dead stain FVS700 (Table S1). After staining, markers were measured on a CytoFlex cytometer (Beckman Coulter, Brea, USA, RRID: SCR_017217). Gating strategy is shown in Figure S2 and described in supplementary methods, where gates were determined by fluorescence minus one (FMO) method (Feher et al., 2014).

During differentiation, flow cytometry was performed at day 10 and 17 of culture (day 0 and 7 of differentiation, Figure 1B). Differentiated cells were stained with monoclonal antibodies CD16, HLA-DR, CD10, CD14, CCR2, CD45, CD11b, CD66b, CD15, and live/dead stain FVS620 (Table S2). Gating strategy is shown in Figure S3 and described in supplementary methods, where gates were determined by FMO method as previously mentioned. Samples were analyzed with FlowJo v10.8 Software (BD Life Sciences).

### HPC-derived monocyte restimulation

After 7 days of differentiation, HPC-derived monocytes were diluted to a concentration of 500,000 cells/mL. A total of 50,000 cells were plated in flat-bottom 96-well plates (Sarstedt) and were stimulated *in duplo* with LPS 10 ng/mL (Sigma-Aldrich, Dt. Louis, MO; *E. coli* serotype 055:B5) further purified as described (Hirschfeld et al., 2000), and Pam3Cys 10 μg/mL (EMC microcollections, Tübingen, Germany; L2000) for 24 h at 37°C with 5% CO_2_. After 24 h, the plate was centrifuged, and supernatant was collected and stored at −20°C until further use. Cytokine assessment of stimulated cells was done using commercial ELISA kits for TNF (DY210), IL-6 (DY201), IL-1β (DY201-05), IL-10 (DY217B), and IL-1RA (DY280) according to the manufacturer (R&D Systems).

### Metabolic analysis (Seahorse)

In a previously hydrated and calibrated cartridge, 100,000 HPC-derived monocytes were plated in quintuplets in Seahorse assay medium (Dulbecco’s modified Eagle’s medium [Sigma] supplemented with 200 mM L-glutamine [Sigma] and 100 mM pyruvate [Sigma]) and incubated for 1 h at 37°C in a non-CO_2_ incubator.

OCR was measured in a XFp Analyzer (Seahorse, Bioscience) in Seahorse medium supplemented with sodium pyruvate 1 mM (Life Technologies), L-glutamine 2 mM (Sigma), and D-glucose 11 mM (Sigma), using a Cell Mito Stress Test Kit (see supplementary methods).

### Pan monocyte MACS isolation

HPC-derived monocytes were isolated after 7 days of differentiation using MACS using a pan monocyte kit according to the manufacturer (MACS, Miltenyi Biotec). After separation, cells were resuspended in IMDM medium + 1% p/s and counted in a CASY Counter.

### RNA isolation for RNA sequencing

After pan monocyte magnetic separation, a total of 5 × 10^5^ cells were transferred to a round-bottom 5 mL polystyrene Falcon tube and were stimulated for 4 h at 37°C, 5% CO_2_ with IMDM only as negative control or LPS 10 ng/mL. After incubation, the cell suspension was centrifuged at 3,420 g, 4°C for 5 min. Pellet was resuspended in RLT buffer, snap-frozen in liquid nitrogen, and stored at −80°C. RNA was isolated using the RNeasy Micro Kit (QIAGEN) according to the manufacturer’s instructions.

### RNA sequencing analysis

RNA quality control was performed using the Bioanalyzer Agilent 2100. Isolated RNA was sent for next-generation sequencing on the DNBSeq 400 platform (BGI Solutions, Hong Kong). Libraries were prepared using the Illumina TruSeq Stranded mRNA kit with a starting input of 100 ng (where available) and sequenced, with the generation of approximately 20 million 100-bp paired-end reads per sample. To infer gene expression levels, RNA sequencing reads were aligned to hg19 human transcriptome using Bowtie2 (Langmead and Salzberg, 2012). Quantification of gene expression, as reads per transcript, was performed using Htseq (Anders et al., 2015), and counts per million (CPM) were calculated. Statistical analysis was performed using DESeq2, with pairwise comparisons performed between IMDM and IL-1β groups. Differentially expressed genes were identified as those showing *p* value < 0.05, FC > 1.5, and CPM >1. Differential gene lists from all comparisons were then merged, and the combined list of differential genes was used for plotting.

### IBIDI flow experiments

IBIDI flow experiments were performed to study EC-monocyte interaction. See supplementary methods for detailed protocol.

### RNA isolation, cDNA synthesis, and qPCR for IBIDI flow experiments

RNA purification of iPSC-derived ECs that encountered HPC-derived monocytes was performed using TriPure (Roche, 11667157001) according to the manufacturer. cDNA was obtained by synthesis using qScript cDNA synthesis kit (QuantaBio 95047-100). Quantitative PCR was done using SYBR green and relevant primers as seen in Table S3 (Integrated DNA Technologies, IDT) in a CFX96 Touch Real-Time PCR (Bio-Rad). For a detailed protocol, see supplementary methods.

### Macrophage differentiation and polarization

At day 20, differentiation and polarization of HPC-derived monocyte-derived macrophages was performed in Roswell Park Memorial Institute 1640 medium (RPMI) supplemented with M-CSF 50 ng/mL (PeproTech, 300-25) for 4 days followed by 3 more days RPMI supplemented with interferon-γ (PeproTech, P01579.1), 50 ng/mL and LPS 10 ng/mL (PeproTech, 297-473-0) for M1 macrophages, and IL-4 10 ng/mL (PeproTech, 130-093-924) for M2 macrophages. Cells were incubated at 37°C, CO_2_ 5%.

### Phagocytosis and quantification

After macrophage differentiation and polarization, a phagocytosis assay was performed according to the manufacturer (Cayman Chemical, 500209), where latex beads (rabbit IgG FITC complexes, green) were added to the macrophage culture for 3 h. Cell nuclei were stained with Hoechst 33342 (1:10,000 in PBS, blue) and dead cells were stained using 4 μM ethidium homodimer-1 (Invitrogen L3224B, red). Phagocytosis rate was quantified as described in supplementary methods.

### Statistical analysis

The experiments present in this article were done using 4 to 6 independent HPC donors (*n* = 4–6). The exact n used is mentioned in detail in the figure legends. Each experiment was performed in duplicate for each independent donor and all the data present in this article are shown as mean ± standard error of the mean. A two-sided value of *p* ≤ 0.05 was considered statistically significant. Statistical analysis was performed using GraphPad Prism version 10.0 (La Jolla, CA, USA). Normality was assessed using Shapiro-Wilk test. Data did not follow a normal distribution; hence, all tests performed were non-parametric unless indicated otherwise per section. For the functional assays of phagocytosis and endothelial cell interactions, we did not perform statistical analysis as this was done only in 2 independent donors.

## Resource availability

### Lead contact

Further information and requests for resources and reagents should be directed to and will be fulfilled by the lead contact, Niels P. Riksen (niels.riksen@radboudumc.nl).

### Materials availability

No new reagents were generated for this article.

### Data and code availability

The accesion number for the RNA-seq data reported in this paper is GEO: GSE253764.

## Acknowledgments

We would like to thank Daniek Kapteijn for helping with IBIDI experiments and Benjamin Cossins for helping with the quantification of the phagocytosis assay. L.A.B.J., M.G.N., and N.P.R. were supported by a CVON grant from the 10.13039/501100002996Dutch Heart Foundation and 10.13039/100018890Dutch Cardiovascular Alliance (CVON2018-27). N.P.R. was further supported by a grant of the ERA-CVD Joint Transnational Call 2018, which is supported by the 10.13039/100002129Dutch Heart Foundation in the Hague (JTC2018, project MEMORY; 2018T093). N.P.R. and M.G.N. were supported by a Project Program Grant of the 10.13039/100000050NHLBI (Project 15-0893 and NIH/NHLBI P01HL131478). S.B. was supported by the 10.13039/100002129Dutch Heart Foundation in the Hague (Dekker grant 2018-T028). M.G.N. was supported by a 10.13039/501100000781European Research Council (ERC) Advanced Grant (FP/2007-2013/ERC grant 2012-322698) and a Spinoza Prize (NWO SPI 92-266). E.L.K. was supported by the 10.13039/501100014470Netherlands Heart Institute (Fellowship #282) and E.L.K. and N.P.R. by the 10.13039/501100014470Netherlands Heart Institute (IMPRESS). B.N. is supported by an 10.13039/501100000925NHMRC (Australia) Investigator Grant (GNT1173314). The figures were created with BioRender.com.

## Author contributions

D.F.-G., S.B., W.H., D.v.E., and N.P.R. were responsible for conceptualization of the study. W.H., D.v.E., N.P.M.S., and W.H.C.R. collected and resourced the bone marrow material. D.F.-G., S.B., and E.L.K. performed the investigation (experiments). Data curation and formal analysis was performed by D.F.-G., S.B., E.L.K., and B.N. Project oversight and supervision was done by S.B., N.P.R., M.G.N., and L.A.B.J. D.F.-G. and S.B. wrote the original draft manuscript, which was afterward reviewed and edited by all the co-authors.

## Declaration of interests

M.G.N. and L.A.B.J. are scientific founders of TTxD and Lemba TX. M.G.N. is scientific founder of Biotrip. W.H.C.R. is a consultant for Stryker for educational purposes only.

## Supplemental information

Document S1. Figures S1–S3, Tables S1–S3, and supplemental experimental procedures

Document S2. Article plus supplemental information
