# Supplementary material for: Interleukin-1β induces trained innate immunity in human hematopoietic progenitor cells in vitro
Source: Stem Cell Reports. 2024 Nov 7;19(12):1651–64. doi: 10.1016/j.stemcr.2024.09.004 (PMC11751800; doi:10.1016/j.stemcr.2024.09.004)
Supplement: Document S1. Figures S1–S3, Tables S1–S3, and supplemental experimental procedures [file mmc1.pdf]

Stem Cell Reports, Volume *19*

Supplemental Information

Interleukin-1β induces trained innate immunity in human hematopoietic progenitor cells *in vitro*

Daniela Flores-Gomez, Willemijn Hobo, Diede van Ens, Elise L. Kessler, Boris Novakovic, Nicolaas P.M. Schaap, Wim H.C. Rijnen, Leo A.B. Joosten, Mihai G. Netea, Niels P. Riksen, and Siroon Bekkering

SUPPLEMENTS

Supplementary Figure 1.

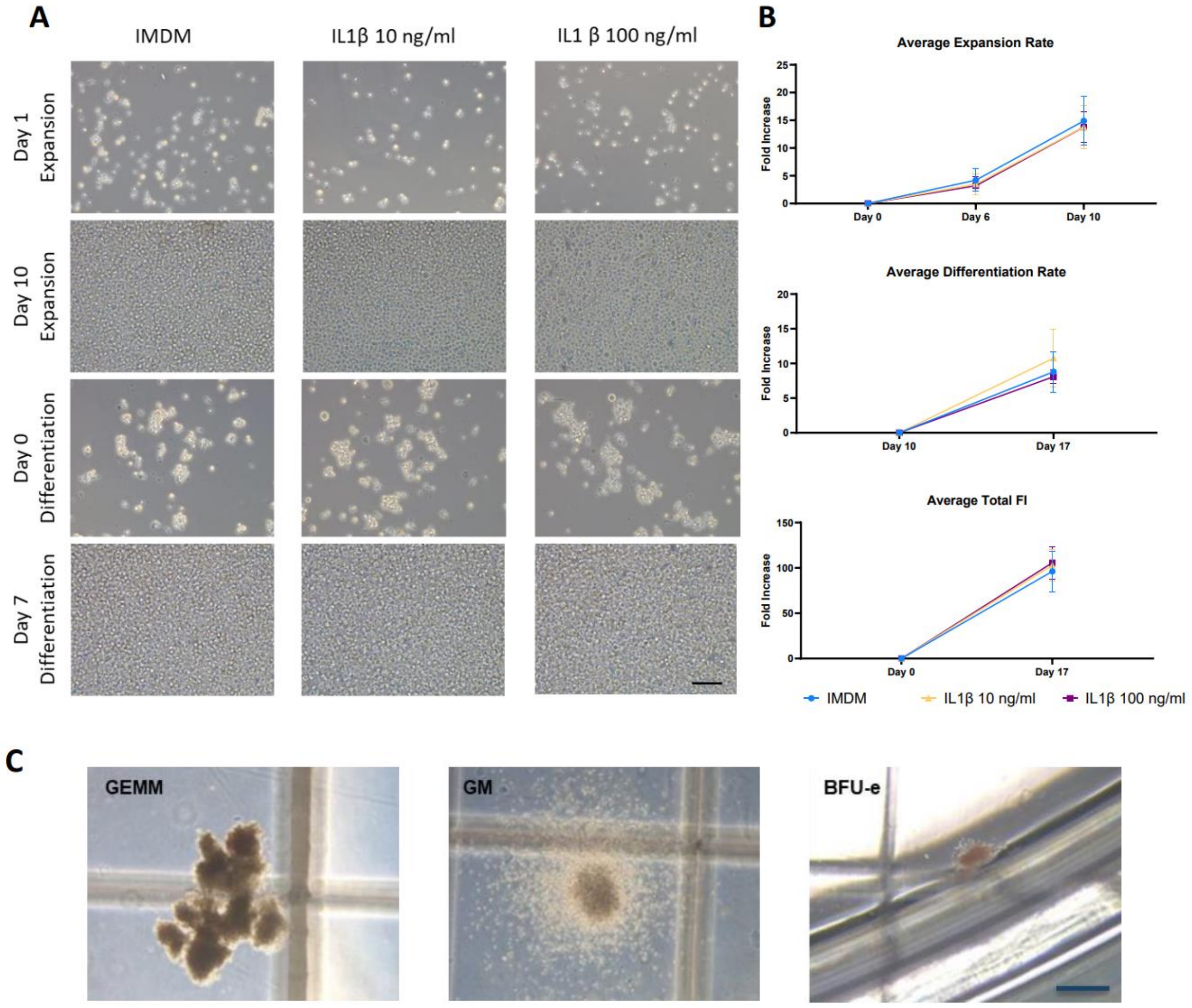

Figure S1, related to figure 1. A) Morphology of HPCs-derived hematopoietic progenitor cells during expansion from day 1 to day 10. At day 10 of expansion, cells were differentiated into monocytes for 7 days with M-CSF (20x magnification, scale bar = 2000 µm). **B)** Average expansion, differentiation and total fold increase during the cell culture time (17 days in total, n=6 independent HPCs donors). **C)** Morphology of HPCs-derived hematopoietic progenitor cells during expansion from day 1 to day 10. At day 10 of expansion, cells were differentiated into monocytes for 7 days with M-CSF (20x magnification, scale bar = 2000 µm).

Supplementary Figure 2.

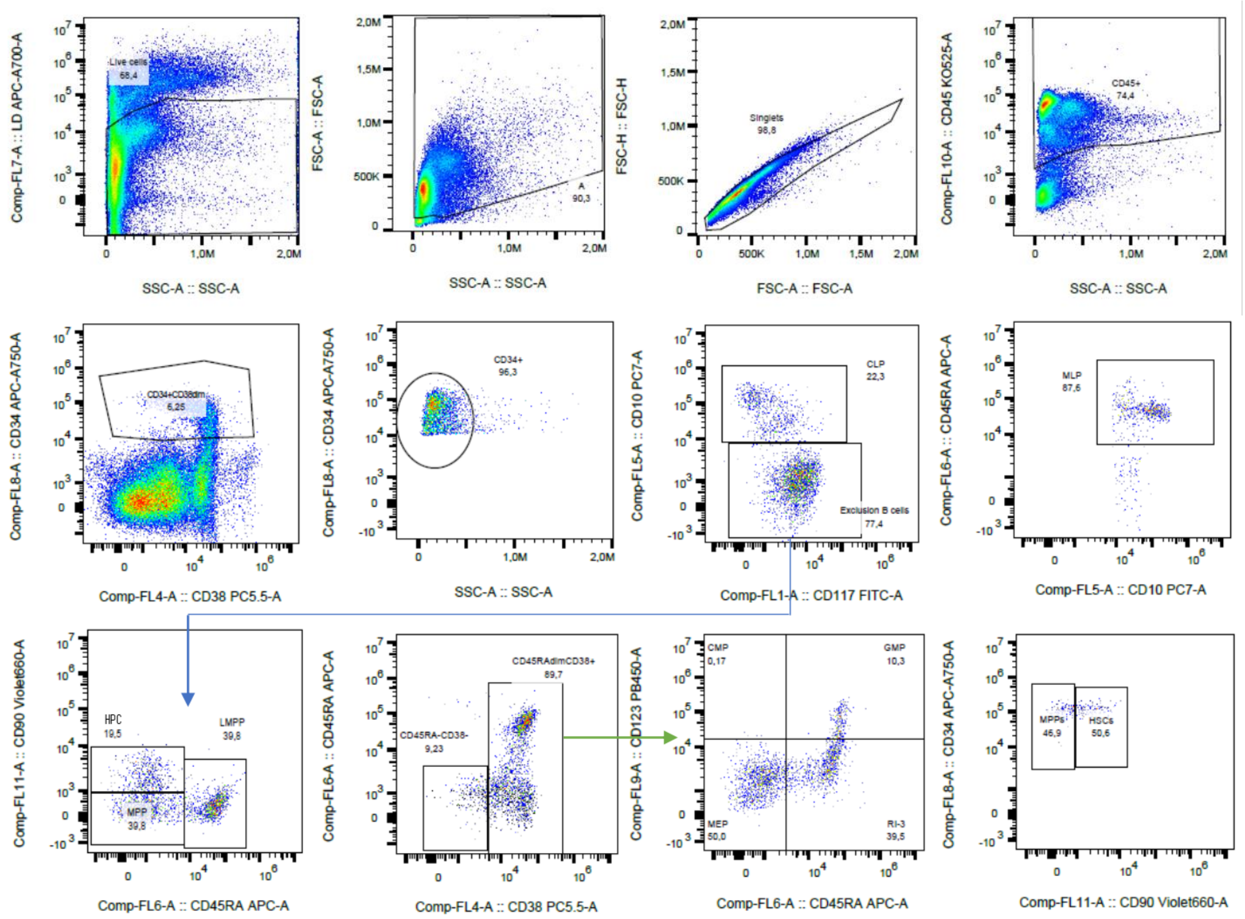

Figure S2. Gating strategy of stem cell progenitor populations from human bone marrow, related to figure 1. Leukocytes were defined as CD45+ cells, after previous exclusion of dead cells, debris and doublets. Stem cells were defined as CD34+CD38dim. Then, the lymphoid line was identified using CD117+CD10+. Next, the non-lymphoid cells were identified using CD117+CD10- where LMPP, MPP and HPC were identified using CD90 and CD45RA. Lastly, in CD45RAdimCD38+ cells, CMP, GMP, R1-3 and MEP were identified using CD45RA and CD123. See table in Figure 3 for additional details. Populations were selected using the fluorescence minus one method (FMO) and side scatter properties.

Supplementary Figure 3

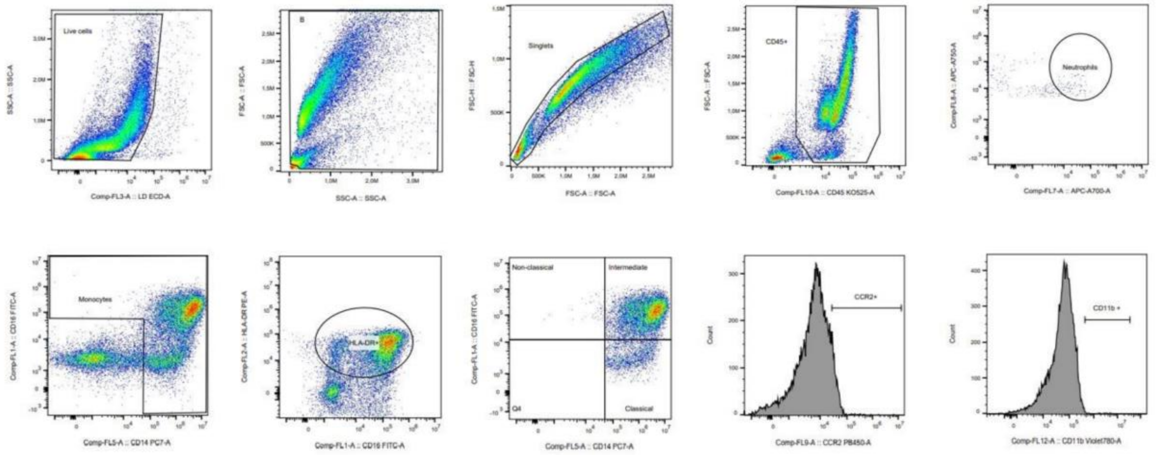

Figure S3. Gating strategy to identify HPCs-derived monocytes, related to figure 1. Leukocytes were defined as CD45+ cells, after previous exclusion of dead cells, debris and doublets. Neutrophils were identified from CD45+ cells using CD66b and CD15. To identify monocytes, CD14 and CD16 markers were used from CD45+, where only HLA-DR+ cells were selected for identification of monocytes subsets. Subsets were defined with CD14 and CD16 as follows: classical (CD14++CD16-), intermediate CD14++CD16+) and non-classical (CD14+CD16++). CCR2 and CD11b markers were also identified from monocytes and its subsets. Populations were selected using the fluorescence minus one method (FMO) and side scatter properties.

Table S1. Flow cytometry panel used to identify stem cell progenitor populations, related to figure 1D. Abbreviations: Cluster of differentiation (CD, fluorescein isothiocyanate (FITC), phycoerythrin-Texas Red conjugate/electron coupled dye (ECD), peridin chlorophyll protein complex (PerCP), phycoerythrin (PE), allophycocyanin (APC).

Marker	Fluorochrome	Clone	Manufacturer	Cat #	RRID #
**CD117**	FITC	104D2	Biolegend	313231	AB_2566218
**CD19**	ECD	J3-119	Beckman Coulter	A07770	AB_2940901
**CD38**	PerCP/Cyanine 5.5 (PC5.5)	HIT2	Biolegend	356613	AB_2562182
**CD10**	PE/Cyanine 7 (PC7)	HI10A	Biolegend	312213	AB_2146549
**CD45RA**	APC	HI100	Biolegend	304112	AB_314416
**CD34**	APC/Fire 750 (APC-A750)	581	Biolegend	343536	AB_2650736
**CD123**	Brilliant violet 421 (BV421)	6H6	Biolegend	306017	AB_10900244
**CD45**	Brilliant violet 510 (BV510)	HI30	Biolegend	304036	AB_2561383
**CD90**	Brilliant violet 650 (BV650)	5E10	Biolegend	328143	AB_2734319

Table S2. Flowcytometry panel used to identify neutrophils, monocyte subsets and monocyte activation markers, related to figure 1E. Abbreviations: Cluster of differentiation (CD), fluorescein isothiocyanate (FITC), phycoerythrin (PE), peridin chlorophyll protein complex (PerCP), allophycocyanin (APC).

Marker	Fluorochrome	Clone	Manufacturer	Cat #	RRID #
**CD16**	FITC	3g8	Biolegend	302006	AB_314206
**HLA-DR**	PE	immu-357	Beckman Coulter	IM1639	AB_131284
**CD10**	PC5.5	HI10	Biolegend	312215	AB_ 10643591
**CD14**	PC7	61D3	LifeTechnologies	25-0149-42	AB_1582276
**CC192 (CCR2)**	BV421	48607	BD Biosciences	564067	AB_2738573
**CD45**	BV510	HI30	Biolegend	304036	AB_2561940
**CD11b**	BV785	ICRF44	Biolegend	301346	AB_2563794
**CD66b**	APC-700	G10F5	Biolegend	305114	AB_2566038
**CD15**	APC-Cy7	W6D3	Biolegend	323047	AB_2750189

Table S3. Overview of the primers used, related to figure 4F. All the primers were ordered from IDT.
Abbreviations: Glyceraldehyde 3-phosphate dehydrogenase (GAPDH), cluster of differentiation (CD), vascular cell adhesion protein-1 (VCAM1), intercellular cell adhesion protein-1 (ICAM1), vascular endothelial growth factor A (VEGFA), von Willebrand factor (vWF) and chemokine ligand 2 (CCL2).

Gene	Protein	Primer name	Primer sequence '5 → 3'
GAPDH	GAPDH	GAPDH Forward	ACACTCACTCTTCTACCTTTG
		GAPDH Reverse	CAAATTCATTGTCGTACCAG
β actin	β actin	β actin Forward	GATCGGCHHCTCCATCCTG
		β actin Reverse	GACTCGTCATACTCCTGCTTGC
CD31	PECAM	CD31 Forward	CATGCAATGAAACCAATAAATGAT
		CD31 Reverse	GAGCCTTCCGTTCTAGAGTATCTG
VCAM1	CD106	VCAM Forward	CATGCAATGAAACCAATAAATGAT
		VCAM Reverse	GAGCCTTCCGTTCTAGAGTATCTG
ICAM1	CD54	ICAM Forward	TTGAACCCCACAGTCACCTAT
		ICAM Reverse	CCTCTGGCTTCGTCAGAATCA
SELP	P-selectin	P-selectin Forward	TGAGCACTGCTTGAAGAAAAAGC

		P-selectin Reverse	CACGTATTCACATTCTGGCCC
SELE	E-selectin	E-selectin Forward	GGCAGTGGACACAGCAAATC
		E-selectin Reverse	TGGACAGCATCGCATCTCA
VEGFA	VEGFA	VEGF Forward	TGCTGTCTTGGGTGCATTGG
		VEGF Reverse	GCATAATCTGCATGGTGATGTTGG
COL1A1	Collagen 1	Collagen Forward	ATCAACCGGAGGAATTTCCGT
		Collagen Reverse	CACCAGGACGACCAGGTTTTC
vWF	vWF	vWF Forward	AGCCTTGTGAAACTGAAGCAT
		vWF Reverse	GCCCTGGTTGCCATTGTAATTC
CCL2	MCP1	MCP1 Forward	GATCGGAACCAAATGAGATCAG
		MCP1 Reverse	GTGGAAAAGGTAGTGGATGC
CD11b	ITGAM	CD11b Forward	ACTTGCAGTGAGAACACGTATG
		CD11b Reverse	TCATCCGCCGAAAGTCATGTG

Supplementary Experimental Procedures

Thawing of MNCs

Per cryovial of cells, a mix of thawing medium containing 1.4 ml heat-inactivated fetal calf serum (FCS, Hyclone Cytiva), 25mM $MgCl_2$, and 66 µg/ml of DNAse (10 mg/ml or 2000 U/mg) was prepared. Cells were taken from liquid nitrogen storage and put in a water bath at 37°C until a small clump of ice was still visible. Cells were transferred to the tube with thawing medium, mixed gently, and incubated for 10 minutes at room temperature. Cells were washed with room temperature PBS and centrifuged for 5 minutes at 500G. Cells were resuspended in IMDM medium and counted in a CASY cell counter (Omni Life Science, OLS).

Flow cytometry

To identify progenitor population, HPCs were selected based on CD45+-CD34+CD38dim after filtering for live cells and singlets. Then, cells from the lymphoid lineage (progenitor B cells) were excluded using CD10-CD117+. Next, CMP, GMP, R1-3 (pre-monocytes), and MEP were identified after gating for CD45RAdimCD38+ using the CD123 and CD45RA markers. Lastly, HPC, LMPP, and MPP were identified from the B cell exclusion using CD90 and CD45RA markers.

To identify bone marrow-derived monocytes, neutrophils were identified based on CD45+CD66b+CD15+ after filtering for live cells and singlets. Next, monocytes were identified based on CD45+HLA-DR+ and side scatter properties. Monocyte subsets were determined using CD14/CD16 as percentage of gated (HLA-DR/CD16). CD11b and C-C chemokine receptor type 2 (CCR2) activation markers were identified in the total monocytes and their subsets by counting the positive signal of the marker based on the FMO. Monocyte subsets were identified according to current recommendations (Thomas et al., 2017).

Metabolic analysis (Seahorse)

First, oligomycin A 1 µM (Sigma) was injected following the basal measurements to inhibit ATP synthase and induce a decrease in electron flow and respiration (ATP-linked respiration and proton leak). Then, carbonyl cyanide-4 (trifluoromethoxy) phenylhydrazone 1 µM (FCCP, Sigma) was injected to cause a

collapse in the proton gradient and alter the mitochondrial membrane potential causing respiration to reach its maximum level (maximal respirator capacity). Lastly, an injection containing antimycin A 2.5 µM (Sigma) and rotenone 1.25 µM (Sigma) is done to shut down mitochondrial respiration and measure respiratory activity outside the mitochondria (reserve capacity).

Extracellular acidification rate (ECAR) was measured in a XFp Analyzer in Seahorse medium supplemented with L-Glutamine 1 mM (Sigma), using a Glyco Stress Test Kit. First, D-Glucose 11 mM is injected to induce the glycolytic pathway under basal conditions (basal glycolysis). Then, oligomycin A 1 µM inhibitor is injected as an ATP synthase inhibitor to switch cellular respiration to glycolysis and measure the cell maximum glycolytic capacity. Lastly, a final injection with 2-deoxy-D-glucose (2-DG, Sigma) 22 mM is performed to inhibit glycolysis resulting.

Interpretation and analysis of Seahorse results was performed for ECAR and OCR measurements according to the guide of Glycolysis and MitoStress Test Kit from the manufacturer (Agilent Seahorse XF).

IBIDI Flow Experiments

A monolayer of iPSC-derived endothelial cells (ECs) was seeded in 0.4 µm IBIDI u-slide (IBIDI 80186) for 2 hours. Then the slide was attached to a pump system (9.3 mbar of pressure, 3 dyn of shear stress, 4.99 ml/min of flow rate, 300/1s of shear rate, 20 s unidirectional and 0.5 s oscillation) for perfusion during 2 hours at 37°C, 5% CO_2. After these 4h, medium was changed to starvation medium containing iPSC-EC medium (Promocell C22110), 0.5% FBS, p/s, and SP431542 (Selleckchem S1067). 20-day differentiated control or 100 ng/ml IL-1β -trained HPC-derived monocytes were added to the perfusion system and flowed through the slide for 2 hours. At the end of the assay, pictures of the slide were taken with an EVOS microscope and adhered monocytes were manually counted by 2 different people. Before the counting, a consensus was reached between the two researchers on the considerations to determine a cell an adhered monocyte or not. The counting was done blinded in at least 3 pictures of the slide. Lastly, iPSCs-derived ECs in the slide were collected and stored in TriZol at -80°C until further use.

RNA isolation, cDNA synthesis, and qPCR for IBIDI flow experiments

RNA purification of iPSCs-derived ECs that encountered HPCs-derived monocytes was performed using TriPure (Roche, 11667157001) and chloroform (Sigma Aldrich 32211-1L-1M) followed by precipitation of RNA with isopropanol (Sigma Aldrich 33539-2.51-M). Isolated RNA was then dissolved in nuclease-free water (Integrated DNA Technologies (IDT) 11-05-01-14) and concentration was measured in Xpose (Trinean, Belgium).

cDNA was obtained by synthesis using qScript cDNA synthesis kit (QuantaBio 95047-100). Quantitative PCR (qPCR) was done using SYBR green and relevant primers as seen in Supplementary Table 3 (Integrated DNA Technologies, IDT) in a CFX96 Touch Real-Time PCR (BioRad). The protocol used consisted of incubation for 3 minutes at 95C, followed by 40 cycles of 10 seconds at 95°C, then 30 seconds at 60°C and lastly 10 seconds at 95°C. Single-product amplification was confirmed using a melting curve analysis. Expression of mRNA was normalized for the geometric mean of the expression of the housekeeping genes human β-actin (ACT2) and glyceraldehyde 3-phosphate dehydrogenase (GAPDH) (ΔCT). Then, relative differences (ΔΔCT) were calculated, and data was used as normalized fold induction (2^ΔΔCT).

Phagocytosis and quantification

Phagocytosis rate was quantified using R 4.2.2 (EBImage package 4.40.0). In short, green, blue, and red channels were brought to a grey scale which was brightened. Then, clusters were separated from the background with adaptive thresholding which were then measure in number of pixels identifying single cells, multiple cells, or debris. The thresholds to calculate single and multiple cells were based on equivalent pixel size of monocytes and macrophages (8 and 10 µm). Clusters were then used to identify green, blue, and red regions and its mean fluorescence. Bead uptake was then calculated based in the overlay of blue and green color.

References:

Thomas, G.D., Hamers, A.A.J., Nakao, C., Marcovecchio, P., Taylor, A.M., McSkimming, C., Nguyen, A.T., McNamara, C.A., and Hedrick, C.C. (2017). Human Blood Monocyte Subsets: A New Gating Strategy Defined Using Cell Surface Markers Identified by Mass Cytometry. Arterioscler Thromb Vasc Biol *37*, 1548-1558. 10.1161/atvbaha.117.309145.
